# Rectal leiomyosarcoma as the initial phenotypic manifestation of Li–Fraumeni-like syndrome: a case report and review of the literature

**DOI:** 10.1186/s13256-022-03671-6

**Published:** 2022-12-19

**Authors:** Natalia Parisi Severino, Jaques Waisberg, Maria Candida Barisson Villares Fragoso, Luiz Guilherme Cernaglia Aureliano de Lima, Flavia Balsamo, Alexandre Cruz Henriques, Bianca Bianco, Flávia de Sousa Gehrke

**Affiliations:** 1 Surgery Department, Hospital do Servidor Público Estadual de São Paulo, São Paulo, SP Brazil; 2 Surgery Department, Faculdade de Medicina do ABC, Santo André, SP Brazil; 3 Teaching and Research Development Center, Hospital do Servidor Público Estadual de São Paulo, São Paulo, SP Brazil; 4 Oncology Department, Instituto do Câncer do Estado de São Paulo, São Paulo, SP Brazil; 5 Pathology Department, Instituto do Câncer do Estado de São Paulo, São Paulo, SP Brazil; 6 Human Reproduction and Genetics Department, Faculdade de Medicina do ABC, Santo André, SP Brazil; 7 Pathology Department, Faculdade de Medicina do ABC, Santo André, SP Brazil

**Keywords:** Leiomyosarcoma, Li–Fraumeni syndrome, *TP53* gene, Mutation, Case report

## Abstract

**Background:**

Leiomyosarcoma is a rare malignant tumor of smooth muscle origin and represents 10–20% of all soft tissue sarcomas. Primary colon and rectal sarcomas constitute < 0.1% of all large bowel malignancies. In Li–Fraumeni syndrome, sarcomas are the second most frequent cancer (25%). Li–Fraumeni syndrome is a genetic disease with a familial predisposition to multiple malignant neoplasms. This syndrome has an autosomal dominant pattern of inheritance and high penetrance characterized by germline *TP53* mutations. Patients with a history of cancer who do not meet all the “classic” criteria for Li–Fraumeni syndrome are considered to have Li–Fraumeni-like syndrome. To the best of our knowledge, this article is the first report of a patient with rectal leiomyosarcoma as the initial phenotypic manifestation of Li–Fraumeni-like syndrome. The authors also present a literature review.

**Case presentation:**

A 67-year-old Brazilian woman underwent anterior rectosigmoidectomy and panhysterectomy secondary to rectal leiomyosarcoma. She subsequently developed carcinomatosis and died 2 years after the operation. Her family medical history consisted of a daughter who died at 32 years of age from breast cancer, a granddaughter diagnosed with adrenocortical carcinoma at 6 years of age and two siblings who died from prostate cancer. A genetic study was carried out to identify a pathogenic variant of Li–Fraumeni syndrome. In the DNA extracted from the peripheral blood leukocyte, restriction fragment length polymorphism was analyzed to search for mutations in the *TP53* gene. The DNA sequencing identified the germline pathogenic variant p. R337H heterozygous in exon 10 of *TP53*. The patient was classified as having Li–Fraumeni-like syndrome.

**Conclusion:**

In patients with rectal leiomyosarcoma, it is advisable to investigate the family history of cancer and perform genetic studies to screen for Li–Fraumeni syndrome.

## Background

Leiomyosarcoma (LMS) is a rare malignant tumor with exclusive smooth muscle differentiation and accounts for 10–20% of all soft tissue sarcomas [1]. LMS accounts for more than 90% of primary colorectal sarcomas [2] but less than 0.1% of malignant colorectal neoplasms. One of the most studied risk factors for the onset of sarcomas is the genetic susceptibility present in Li–Fraumeni syndrome (LFS) [3–5]. LFS is a genetic disease with a familial predisposition to multiple malignant neoplasms, with an autosomal dominant pattern of inheritance and high penetrance, characterized by pathogenic germline variants of the *TP53* gene [6, 7].

Observations of the apparent phenotypic heterogeneity of LFS have been used to propose definitions for families with an extensive history of cancer that does not meet all the “classic” LFS criteria. Individuals from these families have been identified as having Li–Fraumeni-like syndrome (LFLS) [8–11]. The authors describe the case of a patient with LFLS who presented rectal LMS as the first phenotypic manifestation of the syndrome along with a literature review. To the best of our knowledge, this article is the first report of this unusual manifestation of LFLS.

## Case presentation

A 67-year-old Brazilian woman, a retired maid, born in São Paulo (Brazil), experienced pain for 12 months during bowel movements associated with tenesmus, hematochezia, increased bowel movements, and weight loss. The patient was admitted to Hospital Estadual Mario Covas (Santo André, São Paulo, Brazil).

The patient has had one pregnancy and one delivery of a daughter who died at 32 years of age from breast cancer. The patient’s family history also includes an 8-year-old granddaughter with adrenocortical carcinoma (ACC) undergoing treatment for 2 years and two siblings who died from prostate cancer, one at 65 years of age and the other at 68 years of age.

The patient had no addictions to cigarettes, alcohol, or drugs. She had systemic arterial hypertension, hypothyroidism, and osteopenia and was using orally hydrochlorothiazide 25 mg, levothyroxine 37.5 mg, and alendronate 10 mg once a day.

At hospital admission, the patient was hemodynamically stable, and did not present abnormalities on physical or neurological examination and had a BMI of 20.7 kg/m^2^. Digital rectal examination indicated a tumor in the anterior wall of the rectum, 7 cm away from the anal margin. At anoscopy and proctosigmoidoscopy, the appearance of the tumor was vegetating and infiltrating. A colonoscopy revealed the lesion was friable and bleeding to the touch of the colonoscope, with an ulcerated, indurated, cranial extension of 6.5 cm that compromised 50% of the circumference and 80% of the lumen of the organ but did not prevent the progression of the colonoscope up to the cecum. The other segments of the large bowel did not show abnormality. A biopsy of the rectal lesion revealed a mesenchymal pattern of neoplasm.

**Fig. 1:**
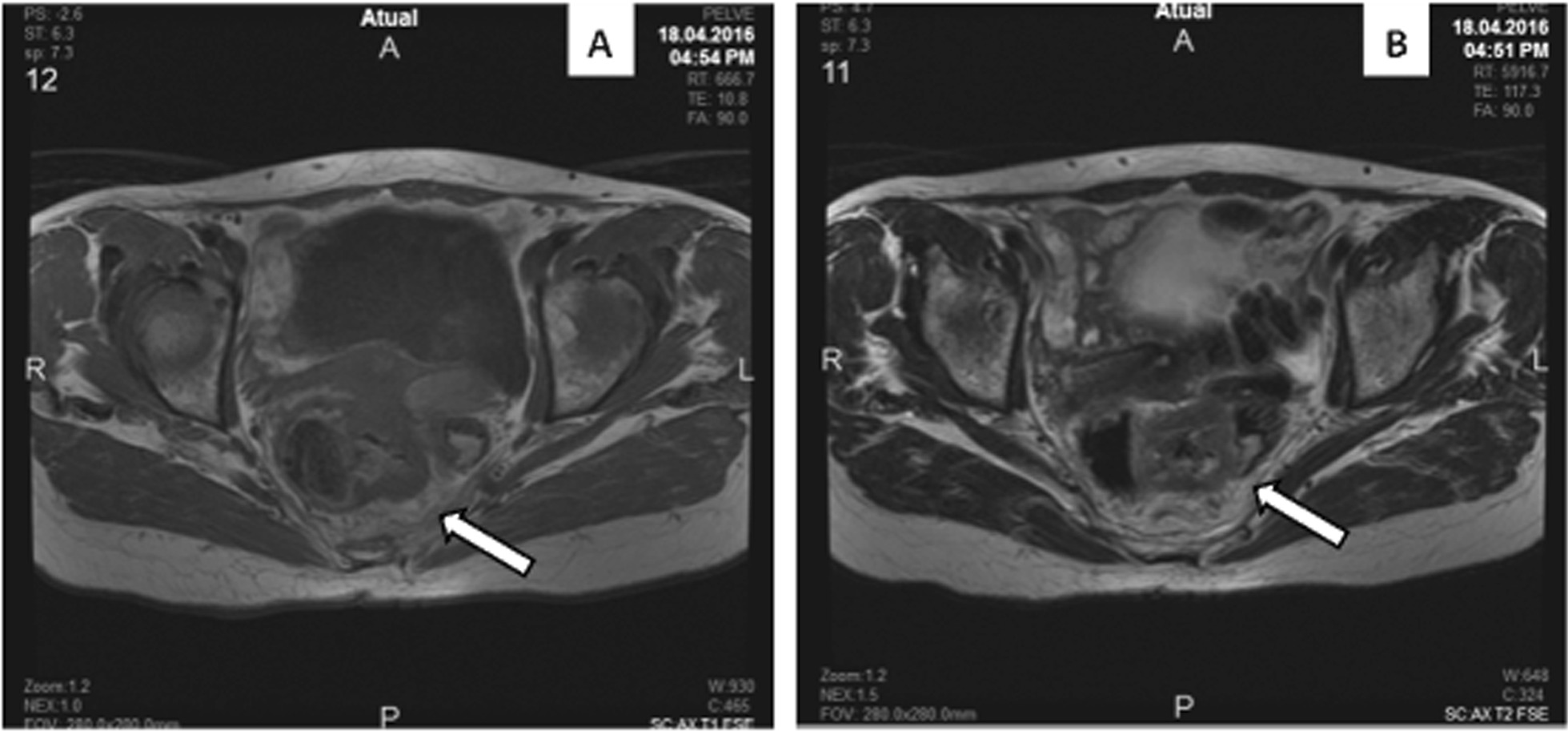
Nuclear magnetic resonance of the pelvic region with an expansive mass in the rectum (shown by arrows). Cross-sections of T1 (**A**) and T2 (**B**)

Computed tomography (CT) of the abdomen and pelvis showed thickening from the middle rectum to the rectosigmoid transition, without liver nodules or alterations in other abdomen organs. A chest CT showed no abnormalities. Pelvic magnetic resonance imaging revealed thickening of the rectum walls 7 cm from the anal border, with suspicion of uterine invasion (Fig. 1). The plasma level of carcinoembryonic antigen (CEA) was normal.

**Fig. 2:**
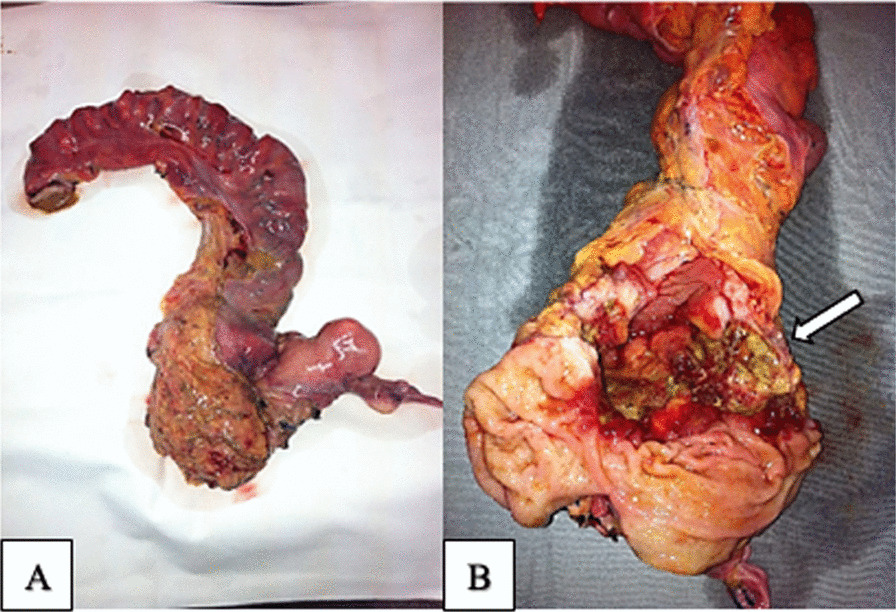
Surgical specimen of en bloc resection of anterior rectosigmoidectomy and panhysterectomy for rectal leiomyosarcoma. **A** Macroscopic aspect of the surgical specimen. **B** Open rectum, where the arrow indicates the tumor

Owing to the clinical manifestations and anatomopathological and radiological findings, it was determined that an oncological resection of the rectal mass was indicated, rather than just a bypass colostomy or neoadjuvant therapy. We performed a laparotomy, and there was no free intraperitoneal fluid, liver metastases, or peritoneal carcinomatosis in the abdominal cavity inventory. The main finding was a voluminous medium rectal mass perforated and blocked anteriorly by the body of the uterus and left adnexa. During the pelvic manipulation for en bloc oncological resection of the affected structures, there was inadvertent unblocking in the tumor area, surgical specimen fracture, and fecal content leakage into the abdominal cavity. As a result of this, a rectosigmoidectomy with colorectal anastomosis was considered a high risk for anastomotic leak, and the patient underwent an anterior rectosigmoidectomy with terminal colostomy using the Hartmann technique and en bloc panhysterectomy owing to adhesions of the body of the uterus and the left ovary in the rectal mass (Fig. 2).

Macroscopic examination of the surgical specimen revealed a hardened, irregular, and ulcerated lesion at the rectum, for which the longest axis was 6.5 cm in length (Fig. 2).

**Fig. 3:**
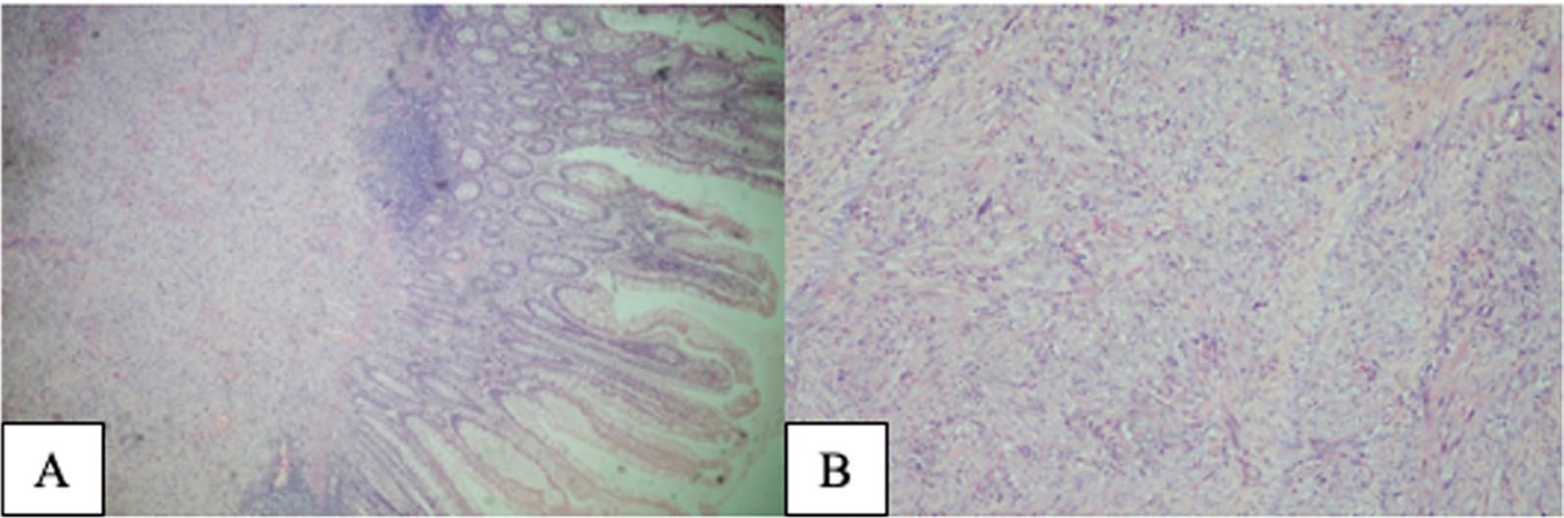
Photomicrographs of leiomyosarcoma. **A** Infiltrative atypical spindle cell neoplasm in colic mucosa *lamina propria* (hematoxylin and eosin, ×40). **B** Neoplasm with sparse atypical cells, bizarre nuclei, hyperchromasia, and pleomorphism (HE, ×100)

Microscopic examination showed an atypical spindle cell proliferation with myoid features, with some cytoplasmic clearing and frequent fasciculation with long bundles, which extrinsically compromised even the deep portion of the mucosa in foci. Cytological atypia was observed, which was represented by enlarged nuclei and sometimes hyperchromatic or bizarre, and there were few pleomorphic nuclei (Fig. 3). The mitotic index was nine mitotic figures per 10 high-power fields (HPFs) (400×/0.5 mm diameter, 4.59/mm^2^), with no atypical forms. There was coagulative-type necrosis, with fibrohyaline replacement, corresponding to 5% of the tumor volume. Neural structures were confined in the tumor without vascular and perineural invasions. The tumor infiltrated the rectal wall up to the perirectal adipose tissue. The surgical resection margins and 16 identified lymph nodes were free of neoplasia. The gynecological organs were not infiltrated by neoplasia. The histological classification based on sarcomas involving abdominal viscera was T2bN0M0 [TNM Classification of Malignant Tumors, 8th Edition/American Joint Committee on Cancer (AJCC)] [12].

**Fig. 4:**
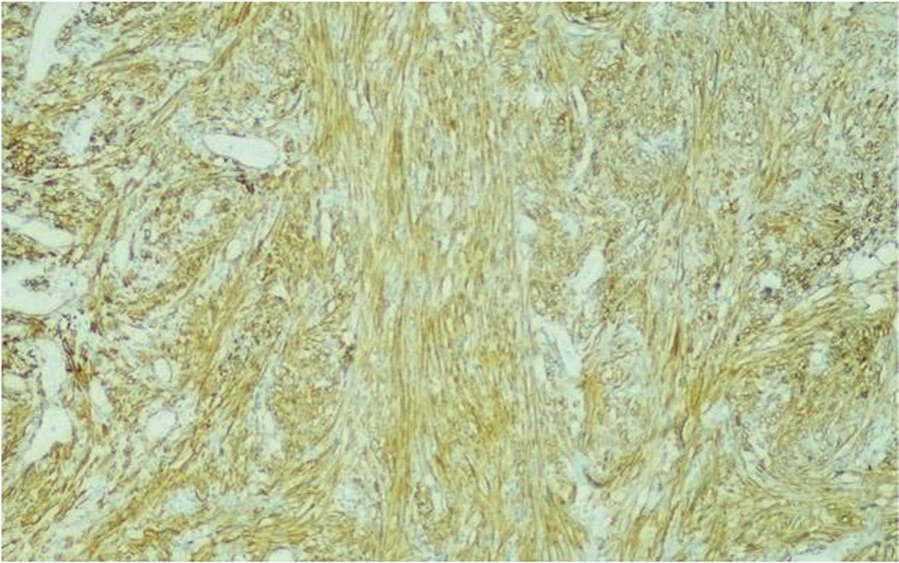
Photomicrograph. Leiomyosarcoma: positive cells strongly and diffusely positive for the smooth muscle marker h-Caldesmon (immunohistochemistry, ×400)

The immunohistochemical study showed positivity for markers such as smooth muscle actin (SMA), beta-catenin (membrane pattern), h-Caldesmon (strong and diffuse) (Fig. 4), estrogen receptor (low intensity), vimentin, and Ki-67 (20% to 30% of the cells of interest). The markers desmin, epithelial membrane antigen (EMA), protein S100, CD34, CD117 (c-kit), and podoplanin D2–40 were not observed in the tumor tissue. The immunohistochemical profile and the anatomopathological findings for the rectal neoplasm were compatible with sarcoma with smooth muscle differentiation, classified as intermediate grade/grade 2 LMS.

The DNA was extracted from the peripheral blood leukocyte for the genetic study using a genomic DNA isolation kit (Gentra System, Minneapolis, MN, USA).

The search for the variant p. R337H was performed by polymerase chain reaction followed by enzymatic digestion (restriction fragment length polymorphism, RFLP). The amplification product for the study of *TP53* exon 10 resulted in a 330-bp fragment. Enzymatic digestion using the restriction enzyme Hhal was performed to recognize the 5′CGC/C3′ sequence in the wild-type exon sequence. After digestion, using the agarose gel electrophoresis, the variant genotype presented three fragments (330 bp, 170 bp, and 160 bp), confirming the presence of the germline pathogenic variant p. R337H in heterozygosis (GA), which was also confirmed in the patient’s daughter and granddaughter.

The patient had an uneventful postoperative course. One year later, she obtained a CT scan of the chest, abdomen, and pelvis, revealing multiple liver, lung, and peritoneal metastases. Consequently, the patient received first-line chemotherapy initially with six cycles of intravenous doxorubicin (60 mg/m^2^), followed by two more cycles of doxorubicin with a reduced dose (57.5 mg/m^2^) owing to a mild neutropenia (1230/mm^3^) developed after the sixth cycle. There were no other laboratory changes in the blood count, liver, or kidney function. The second-line chemotherapy was with six cycles of intravenous gemcitabine (800 mg/m^2^) and docetaxel (30 mg/m^2^). Initially, there was a reduction in the dimensions of the nodules scattered through the lungs, liver, and peritoneum. However, the metastases increased in size and number, and the patient was no longer submitted to disease-modifying therapy proposals. The patient was admitted with persistent and severe anal pain and neoplastic cachexia under the oversight of a palliative care team. For pain control she was taking orally dipyrone 1 g every 6 h, morphine 10 mg every 4 h, and methadone 5 mg every 12 h. The patient died 2 years after the rectal tumor resection.

## Discussion and conclusions

This article presents a case of a 67-year-old woman with a germline pathogenic variant of *TP53,* classified as having LFLS owing to a rectal leiomyosarcoma and a family medical history compatible with the syndrome. To the best of our knowledge, this article is the first report of a rectal LMS as a phenotypic manifestation in a patient with LFLS.

LMS in the large bowel is mainly found in individuals between 50 and 70 years old [1], which is similar to the patient’s age in this report, and there is no prevalence between the sexes [1, 4, 13]. The clinical findings for rectal LMS are the same as those for rectal adenocarcinoma [2, 14, 15]. Both can manifest as bleeding, change in bowel habits, tenesmus, obstruction, and perforation [14, 15]. Rectal LMS is often detected by palpation in a digital rectal examination, as 80% of the tumors are located in the distal third of the rectum and generally grow into the lumen [2, 14, 15], as was observed in this report.

Colonoscopy with biopsy of the lesion remains the most important diagnostic modality. LMS originating in the colon and rectum are relatively avascular, noncapsular, well-circumscribed lesions, ranging from 5 cm to 15 cm in diameter [14–16], as observed in the present case. Histologically, these lesions are characterized by intertwined bundles of infiltrative atypical spindle smooth muscle cells, cellular pleomorphism, and eosinophilic cytoplasm, frequently with bizarre nuclei, including giant cells [3, 13]. LMS are generally high grade, with high mitotic activity and anaplasia. Although lymphatic spread has been reported, these tumors mainly spread via the direct route to neighboring organs and the hematogenous pathway to distant organs [1, 3, 15, 16], especially to the liver, lungs, and peritoneum [1, 13], as was observed in the patient in this report.

The frequency of mitosis (5–20 mitoses per 10 HPF) is the most helpful indicator of the malignant potential of LMS to develop local recurrences and distant metastases [2, 13–15]. However, there are reports of LMS that developed metastases even with minimal atypia [1–3, 13]. In the case presented in this report, the frequency of mitosis was nine mitoses per 10 HPF.

The differential diagnosis for LMS includes submucosal tumors such as leiomyoma, gastrointestinal stromal tumor (GIST), and inflammatory fibroid polyp. LMS can be distinguished from leiomyoma, its benign congener, by larger and atypical tumor cells, higher proliferative index, atypical mitotic figures, and pleomorphism. LMS is immunohistochemically distinct from other mesenchymal tumors owing to the positive and diffuse expression of SMA, MSA, desmin, or h-Caldesmon-specific muscle actin (MSA–HHF35). Some studies show that actin and h-Caldesmon can be more sensitive than desmin in detecting smooth muscle malignancies [17]. In the present report, the desmin was not observed in the tumor tissue. Negativity of gastrointestinal stromal tumor markers (CD117, CD34, and DOG1) or schwannoma markers (S100 protein) was also observed in the case described [1–3, 16].

There is no defined treatment strategy for rectal LMS owing to the limited data, but surgical treatment remains the primary therapeutic approach [15, 16]. Curative surgical resection of the primary LMS can be achieved in 50% to 60% of cases [2, 14–16]. Although gastrointestinal LMS lymph node metastases are relatively uncommon, lymph node removal is advisable when not overly invasive [2, 14, 15]. Local rectal resection is recommended for selected cases of LMS with tumor diameters < 2 cm, patients at high risk for anesthesia, and patients with tumors with a low degree of malignancy [2, 14]. Similar to other sarcomas, LMS is relatively chemoresistant and radioresistant [2, 14–16].

Studies suggest that LMS is highly aggressive with a poor prognosis, but a complete surgical resection can improve the prognosis [13–15]. Although the prognostic factors are not yet apparent, a tumor diameter ≥ 5 cm is considered an unfavorable prognostic parameter [13–15]. Hematogenous dissemination to the liver and lungs is described as the most frequent cause of death in these patients [2, 14, 15]. The patient in the present report had a tumor with a 6.5-cm diameter and died with liver, pulmonary, and peritoneal metastases.

The “classic” LFS condition is characterized by the development of soft tissue sarcoma before 45 years of age in an individual with a first-degree relative with cancer before 45 years of age and a first- or second-degree relative with any cancer before 45 years of age or with sarcoma at any age group [8, 18, 19]. Furthermore, the *TP53* mutation should also be investigated in the following situations (Chompret criteria): the presence of a characteristic tumor of the LFS spectrum before 46 years of age and at least one first- or second-degree relative with a typical LFS neoplasm before age 56 or with multiple tumors; the presence of various tumors (other than multiple breast neoplasms) in the same individual, two of which belong to the LFS spectrum and the first of which occurs before 46 years of age; the presence of ACC or choroid plexus tumor, regardless of family history; women with a personal history of breast cancer before 36 years of age and who are negative for the pathogenic variants in *BRCA1* and *BRCA2* [8, 19, 20].

Birch *et al*. [21] observed that less than half of the families with *TP53* mutations meet the “classic” criteria for the clinical diagnosis of LFS [22], and defined additional criteria for the syndrome: an individual diagnosed with childhood cancer or sarcoma, brain tumor, or ACC before 45 years of age and first- or second-degree relative with LFS-spectrum cancer (osteosarcoma or soft tissue sarcoma, breast cancer, brain tumor, leukemia, ACC, melanoma, prostate cancer, and pancreatic cancer) at any age and first- or second-degree relative with any cancer before 60 years of age [21].

Eeles [23] proposed more comprehensive criteria for including families in LFS: at least two first- or second-degree relatives of any age diagnosed with LFS-spectrum cancer. Subsequently, Eeles [23] included individuals with sarcoma at any age and at least two other tumors diagnosed in one or more first- or second-degree relatives: breast cancer before 50 years of age, LFS-spectrum cancer before 60 years, or a sarcoma at any age [24]. These criteria are known as LFLS. *TP53* mutations are detected in 14% to 40% of families that meet the criteria for LFLS [18, 25].

In Brazil, the high prevalence of LFS is mainly related to a specific pathogenic variant p. Arg337His (*TP53*: c.1010G>A, p. Arg337His) in exon 10 of the *TP53* gene, also known as p. R337H [8, 26]. The pathogenic variant p. R337H has low penetrance but a high prevalence in Brazil’s southern and southeastern regions [27], where the patient was born and lived.

The patient in the present report had the phenotypic manifestation (rectal LMS) at 67 years of age. Furthermore, the patient had two first- and second-degree relatives (a daughter and a granddaughter, respectively) who had been diagnosed with cancer (breast before 50 years of age and adrenal gland before 60 years of age, respectively). In addition, the patient had two brothers who had died of prostate cancer. Therefore, the patient was considered to have criteria for clinical diagnosis of LFLS (Table 1). Owing to this, we investigated the *TP53* mutation, and the patient was shown to have the pathogenic variant p. R337H in heterozygosity in exon 10 of *TP53.*

**Table 1:** LFS criteria and LFLS definitions

LFS—classic criteria [8, 18, 19]	• Soft tissue sarcoma before 45 years of age *and* • First-degree relative with cancer before 45 years of age *and* • A first- or second-degree relative with any cancer before 45 years of age or a sarcoma at any age
LFS—Chompret criteria [8, 19, 20]	• Tumor of the LFS spectrum before 46 years of age • *and* at least one first- or second-degree relative with a typical LFS neoplasm before age 56 or with multiple tumors *or* • The presence of various tumors (other than multiple breast neoplasms) in the same individual, two of which belong to the LFS spectrum and the first of which occurs before 46 years of age *or* • The presence of ACC or choroid plexus tumor, regardless of family history *or* • Woman with a personal history of breast cancer before 36 years of age and who are negative for the pathogenic variants in *BRCA1* and *BRCA2*
LFLS—Birch definition [21]	• An individual diagnosed with childhood cancer, sarcoma, brain tumor, or ACC before 45 years of age *and* • First- or second-degree relative with LFS spectrum cancer at any age *and* • First- or second-degree relative with any cancer before 60 years of age
LFLS—Eeles definition [23]	• At least two first- or second-degree relatives of any age, diagnosed with LFS-spectrum cancer *or* • Sarcoma at any age • *and* at least two other tumors diagnosed in one or more first- or second-degree relatives: breast cancer before 50 years of age, LFS-spectrum cancer before 60 years, or a sarcoma at any age

*LFS* Li-Fraumeni syndrome,* LFLS* Li-Fraumeni-like syndrome,* ACC* Adrenocortical carcinoma

Thus, patients with rectal LMS may be carriers of LFS or LFLS, and a targeted investigation of family history of cancer and a genetic study should be performed. Genetic counseling can contribute to the diagnosis of this syndrome. These patients and their families should be monitored for the prevention or early diagnosis of other tumors that they are predisposed to during their lifetime. Genetic screening tests for suspected LFS populations of reproductive age could be used to identify individuals carrying mutations. Thus, it would be possible to prevent the transmission of the mutated allele to offspring through genetic evaluation of embryos (preimplantation genetic test) produced by in vitro fertilization. For patients who have already been diagnosed with LFS or LFLS, a routine proctological exam and colonoscopy are essential tests for screening for sporadic colorectal carcinomas and rectal LMS because diagnosis and early treatment can change the clinical course of this unusual neoplasm. The major limitation of this case report is associated with the use of retrospective medical record information. Future studies are required to identify the incidence and prevalence of rectal LMS in patients with LFS and LFLS.

## Data Availability

The datasets used and analyzed during the current study are available from the corresponding author on reasonable request.

## Abbreviations

ACC: Adrenocortical carcinoma
AJCC: American Joint Committee on Cancer
CT: Computed tomography
CEA: Carcinoembryonic antigen
dNTPs: Phosphated deoxyribonucleotides
EMA: Epithelial membrane antigen
GIST: Gastrointestinal stromal tumor
HPFs: High-power fields
IARC: International Agency for Research on Cancer
LFS: Li–Fraumeni syndrome
LFLS: Li–Fraumeni-like syndrome
LMS: Leiomyosarcoma
MSA: Specific muscle actin
RFLP: Restriction fragment length polymorphism
SMA: Smooth muscle actin
